# Development and Validation of an Automatic System for Intracerebral Hemorrhage Medical Text Recognition and Treatment Plan Output

**DOI:** 10.3389/fnagi.2022.798132

**Published:** 2022-04-08

**Authors:** Bo Deng, Wenwen Zhu, Xiaochuan Sun, Yanfeng Xie, Wei Dan, Yan Zhan, Yulong Xia, Xinyi Liang, Jie Li, Quanhong Shi, Li Jiang

**Affiliations:** ^1^Department of Neurosurgery, The First Affiliated Hospital of Chongqing Medical University, Chongqing, China; ^2^School of Intelligent Technology and Engineering, Chongqing University of Science and Technology, Chongqing, China

**Keywords:** stroke, intracerebral hemorrhage (ICH), natural language processing (NLP), artificial intelligence (AI), neurosurgery emergency electrical medical record database (N-eEMRD)

## Abstract

The main purpose of the study was to explore a reliable way to automatically handle emergency cases, such as intracerebral hemorrhage (ICH). Therefore, an artificial intelligence (AI) system, named, H-system, was designed to automatically recognize medical text data of ICH patients and output the treatment plan. Furthermore, the efficiency and reliability of the H-system were tested and analyzed. The H-system, which is mainly based on a pretrained language model Bidirectional Encoder Representations from Transformers (BERT) and an expert module for logical judgment of extracted entities, was designed and founded by the neurosurgeon and AI experts together. All emergency medical text data were from the neurosurgery emergency electronic medical record database (N-eEMRD) of the First Affiliated Hospital of Chongqing Medical University, Chongqing Emergency Medical Center, and Chongqing First People’s Hospital, and the treatment plans of these ICH cases were divided into two types. A total of 1,000 simulated ICH cases were randomly selected as training and validation sets. After training and validating on simulated cases, real cases from three medical centers were provided to test the efficiency of the H-system. Doctors with 1 and 5 years of working experience in neurosurgery (Doctor-1Y and Doctor-5Y) were included to compare with H-system. Furthermore, the data of the H-system, for instance, sensitivity, specificity, accuracy, positive predictive value (PPV), negative predictive value (NPV), and the area under the receiver operating characteristics curve (AUC), were calculated and compared with Doctor-1Y and Doctor-5Y. In the testing set, the time H-system spent on ICH cases was significantly shorter than that of doctors with Doctor-1Y and Doctor-5Y. In the testing set, the accuracy of the H-system’s treatment plan was 88.55 (88.16–88.94)%, the specificity was 85.71 (84.99–86.43)%, and the sensitivity was 91.83 (91.01–92.65)%. The AUC value of the H-system in the testing set was 0.887 (0.884–0.891). Furthermore, the time H-system spent on ICH cases was significantly shorter than that of doctors with Doctor-1Y and Doctor-5Y. The accuracy and AUC of the H-system were significantly higher than that of Doctor-1Y. In addition, the accuracy of the H-system was more closed to that of Doctor-5Y. The H-system designed in the study can automatically recognize and analyze medical text data of patients with ICH and rapidly output accurate treatment plans with high efficiency. It may provide a reliable and novel way to automatically and rapidly handle emergency cases, such as ICH.

## Introduction

Spontaneous intracerebral hemorrhage (ICH) is a kind of non-traumatic hemorrhage in the brain parenchyma (Qureshi et al., 2001). It is a common emergency in neurosurgery with high morbidity, disability, and mortality (Broderick et al., 2007). With an incidence rate of 12–15/10 million, ICH has traditionally lagged behind ischemic stroke (Hemphill et al., 2015). In western countries, ICH accounted for as high as about 15% of all strokes and 10–30% of all hospitalized patients with stroke (Steiner et al., 2014). In China, the situation is even worse due to the huge population, and ICH accounted for about 18–47.6% of all strokes in China, and the mortality in 30 days is up to 35–52%, and only about 20% of patients were able to recover their self-care ability after 6 months (Wu et al., 2019; Zhou et al., 2019). Therefore, a timely and proper treatment, which bases on an accurate analysis of the patient’s condition, is crucial and may significantly influence the prognosis of patients with ICH (Sangha and Gonzales, 2011; Al-Kawaz et al., 2020).

With the expansion of computational power and information content in medical data, brilliant progress has been made in the automatic interpretation of image and text data (Höller et al., 2017; Afzal et al., 2018; Chilamkurthy et al., 2018; Beheshti et al., 2020; Kim et al., 2020; Parthasarathy et al., 2020; Teng et al., 2021). Recently, novel machine learning-based algorithms were developed to segment and interpret the CT image of patients with ICH, hoping to provide accurate and automated treatment (Ironside et al., 2019, 2020; Nawabi et al., 2020; Zhou et al., 2020). Although the interpretation of CT image provides objective and vital information about the intracranial condition, other clinical information, for instance, medical history and physical examination, is also essential for the treatment of ICH (Brott et al., 1986; Liao et al., 2015; Velupillai et al., 2018; Wang et al., 2018; Sung et al., 2020; Zhou et al., 2020). However, most algorithms focus on image interpretation, and medical text interpretation attracts far less attention.

The aim of this study was to explore a reliable method to rapidly analyze the medical text data of ICH patients and make a treatment plan. To achieve this aim, we designed a system to automatically analyze the medical text data of patients with ICH (such as the medical history, physical examination, and CT report) and provide a treatment plan. Furthermore, agreement analysis for the treatment plan retrieved from the algorithm and human doctors was also performed to evaluate the effectiveness of the system.

## Materials and Methods

The study met ethical standards approved by the Ethical Committees of the First Affiliated Hospital of Chongqing Medical University. The cases used in this study consisted of simulated cases and real ICH cases, both of which were from the neurosurgery emergency electrical medical record database (N-eEMRD) of the First Affiliated Hospital of Chongqing Medical University. Each case from the N-eEMRD consists of basic patient information, chief complaint, history of current and past illness, physical examination, and head CT results. The real ICH cases were collected from three medical centers, i.e., First Affiliated Hospital of Chongqing Medical University, Chongqing Emergency Medical Center, and Chongqing First People’s Hospital, from January 2017 to May 2021, and the inclusion criteria of cases were as follows: (1) patients diagnosed with spontaneous cerebral hemorrhage in compliance with the latest guideline of stroke (Hemphill et al., 2015; Cordonnier et al., 2018; Wu et al., 2019) and (2) patients who were aged 10–80 years old. Exclusion criteria were as follows: (1) patients with incomplete medical history or physical examination and (2) no head CT results.

### Data Set and Demographic Characteristics

The cases included in this study were all from N-eEMRD. Among them, 1,000 simulated ICH cases were divided into a training set (700 cases) and a validation set (300 cases). A total of 1,052 real ICH cases were collected from three medical centers, and 68 cases were excluded due to the inclusion and exclusion criteria. Finally, 984 consecutive real ICH cases were recruited as testing sets. The demographic characteristics of all cases are shown in Table 1, and there were no differences in demographic variables among the training set, validation set, and testing set. Values were presented as mean ± standard deviation (SD) or number (column percent) as appropriate.

**TABLE 1:** Baseline characteristics of the total study population.

Characteristic	Training set (*n* = 700)	Validation set (*n* = 300)	Testing set (*n* = 984)	*p*-Value
Age, years	66 ± 15.1	65 ± 14.9	64 ± 15.0	0.763
Female, %	396 (56.6)	176 (58.7)	551 (56.0)	0.758
Male, %	304 (43.4)	124 (41.3)	433 (44.0)	0.736
Weight, kg	61.5 ± 11.7	60.9 ± 12.1	62.1 ± 12.4	0.695
Height, cm	161 ± 14.5	159 ± 15.9	160 ± 15.3	0.786
Hypertension, %	476 (68.0)	219 (73.0)	699 (71.0)	0.758
Diabetes, %	189 (27.0)	84 (28.0)	267 (27.1)	0.774

For simulated ICH cases in the training and validation sets, the treatment plans made by two professors with more than 20 years of experience in neurosurgery were set as the gold standard. While for real ICH cases of testing set, the original treatment plan of the real ICH case was set as the gold standard. The treatment plans were divided into two types: (1) Plan I, emergency surgery, which meant the patient had an indication for emergency surgery and needed immediate emergency surgery. (2) Plan II, non-surgical treatment, there is no indication for emergency surgery, but medication is needed. In addition, Plan II was further divided into Plans IIA and IIB. Plan IIA meant the patients did not have an indication for emergency surgery for the time being, but their condition was not stable and they might need surgery if their conditions deteriorated. Plan IIB meant the patient had no indication for emergency surgery but required drug treatment, and their condition was relatively stable.

All the treatment plans in this study were divided into Plans I and II, Plan II was further divided into Plans IIA and IIB. Therefore, not only the treatment plans provided by both H-system and doctors but also the treatment plans which were set as “golden standard” would be classified as Plans I, IIA, or IIB according to the definition of the treatment plans. Then the treatment plans provided by both H-system and doctors were collected and subsequently compared with the “golden standard,” calculating the data, such as sensitivity, specificity, positive predictive value (PPV), negative predictive value (NPV), and the area under the receiver operating characteristics curve (AUC).

### Preprocessing of Electronic Medical Text

Due to spelling errors, non-text symbols, and abbreviations, narrative clinical medical records retrieved from N-eEMRD require a series of text preprocessing by an experienced neurosurgeon. First, the misspelled phrases were checked and corrected. Second, with concerted efforts of the experienced neurosurgeon and artificial intelligence (AI) experts, keywords from the patient’s medical records have been identified and labeled (Supplementary Appendix 1). For example, keywords of medical history and physical examination included the Glasgow Coma Scale (GCS) score, vital signs (such as heart rate, blood pressure, and oxygen saturation value), consciousness grading, pathological reflex, pupil reflection, and clinical symptoms (headache, nausea, vomiting). Meanwhile, the keywords in the standardized CT report included the location of bleeding, the amount of bleeding, the shape and size of the ventricle, whether the ventricle is cast, and the shift of the midline structure (Supplementary Appendix 2; Chilamkurthy et al., 2018).

### Development of the H-System

**FIGURE 1:**
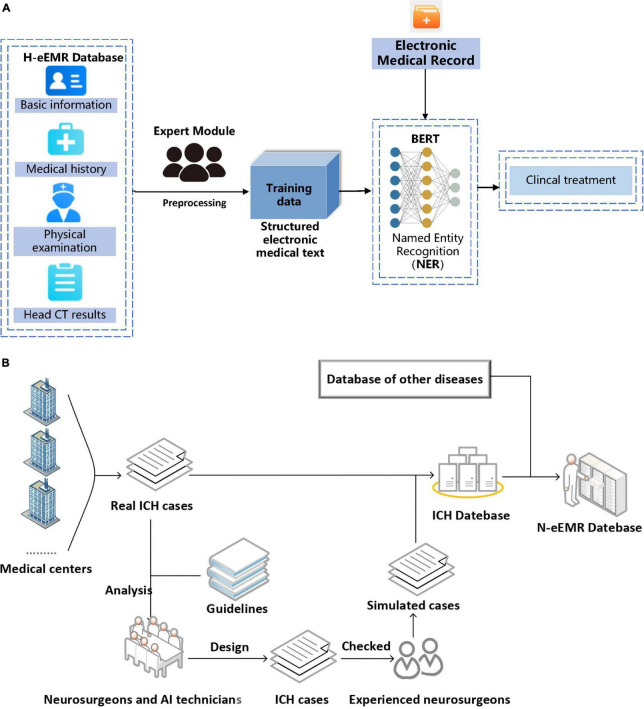
**(A)** Flowchart of H-system. Medical texts were input into H-system and then analyzed. After that, the treatment plan was automatically output. BERT, Bidirectional Encoder Representations from Transformers. **(B)** Found of neurosurgery emergency electronic medical record database (N-eEMRD). Simulated intracerebral hemorrhage (ICH) cases were used for the development of H-system and internal validation with help of an experienced neurosurgeon and reference of the guidelines, and real ICH cases were used for external validation and efficiency testing.

The emergency electrical medical record (eEMRs) of simulated ICH cases were all obtained from the N-eEMRD and randomly divided into training and verifying and testing sets (the ratio is 6:2:2). The training set was used for model fitting, and the validation set was used to validate the accuracy of the model for tuning the parameters of the model, then the testing set was used to evaluate the generalization ability of the model. The H-system consisted of a pretrained language model Bidirectional Encoder Representations from Transformers (BERT) for named entity recognition (NER) (Zhou et al., 2004; Wang et al., 2019; Gligic et al., 2020) and an expert module for logical judgment of extracted entities according to the ICH clinical treatment rules (Figure 1A). Both the logical judgment of extracted entities and the ICH clinical treatment rules were discussed and reached an agreement by the neurosurgeon and AI experts. Moreover, the ICH clinical treatment rules conformed to the latest guidelines for ICH (Figure 1B).

After the ICH patient’s eEMR was input into the system, the medical text was automatically analyzed, and then clinical treatment plans were the output. With the use of the same labels as on the primary data set, the performance of the system was assessed on the independent external-testing data sets for training and verification.

### Performance and Evaluation of the H-System

The H-system was built based on BERT and then was trained and validated by simulated ICH cases. The eEMRs of real ICH cases were used as a testing set to evaluate the performance of the H-system. Furthermore, the eEMRs of real ICH cases were randomly selected and provided to H-system and doctors, both of who made the treatment plans after the analysis of these eEMRs. The doctors were divided into two groups, Doctor-5Y and Doctor-1Y. The Doctor-5Y Group consisted of 3 doctors with 5 years of working experience in neurosurgery, while Doctor-1Y consisted of 3 doctors with 1 year of working experience in neurosurgery.

#### Comparison of Time and Accuracy in Dealing With a Fixed Number of Cases

A total of 60 real cases, all of which were randomly selected from N-eEMRD, were provided to both H-system and doctors. Then the time that H-system and doctors spent on handling these cases was recorded and analyzed. Furthermore, data of H-system and doctors, for instance, sensitivity, specificity, PPV, NPV, and the AUC, were calculated and compared, respectively, by setting the original treatment plan of the real ICH case as a golden standard.

#### Comparison of the Number of Cases and Accuracy in a Fixed Time

To further study the effectiveness of the H-system, the number of cases handled by the H-system and doctors in 60 min were recorded and compared. Furthermore, the differences between H-system and doctors in sensitivity, specificity, accuracy, PPV, and NPV of treatment plans were also analyzed, respectively.

### Statistical Analysis

The SPSS statistical software (Version 26.0 for Windows, IBM Corp., Armonk, NY, United States) was performed for statistical analysis in this study. Categorical variables were expressed as absolute numbers and percentages, continuous variables as mean ± SD or median (95% confidence interval [CI]). In the validation and testing phase, the sensitivity, specificity, PPV, NPV, and AUC were used to evaluate the performance of the H-system. AUC measures the efficiency of different groups. Inter-rater agreement was measured using Cohen’s κ value. Accuracy was calculated to evaluate the performance of the H-system. The two-tailed *p* was considered to be statistically significant when it is <0.05.

## Results

### Efficiency of H-System

#### Overall Efficiency of H-System in a Testing Set

**FIGURE 2:**
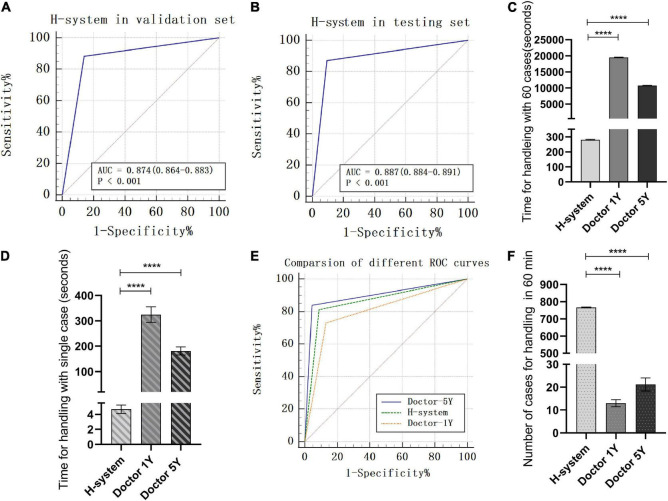
Testing of H-system’s efficiency and reliability. **(A)** ROC of H-system in validation set. **(B)** ROC of H-system in testing set. (**A** and **B** meant that the treatment output by H-system was accurate and reliable.) **(C)** The total time H-system, Doctor-1Y, and Doctor-5Y spent on 60 cases. **(D)** The mean time H-system, Doctor-1Y, and Doctor-5Y spent on single case. (**C** and **D** indicated that the time of Doctor-1Y and Doctor-5Y spent on the fixed quantity cases was significantly longer than H-system.) **(E)** Comparison of ROC for handling 60 cases among H-system, Doctor-1Y, and Doctor-5Y. The AUC of Doctor-1Y was significantly lower than that of H-system. However, a high degree of agreement on treatment plan was found between Doctor-5Y and H-system. **(F)** Comparison of number of cases handled by H-system, Doctor-1Y, and Doctor-5Y in 30 min. The figure means that the number of cases handled by H-system was significantly greater than that of Doctor-1Y and Doctor-5Y in a fixed time. **** means there was a significant statistical difference (*p* < 0.001).

By comparing with the golden standard of simulated ICH cases, the accuracy, specificity, and sensitivity of the H-system in the validation set were 87.00 (86.18–87.82)%, 85.08 (82.86–87.29)%, and 89.58 (86.61–92.55)%, and the AUC value was 0.874 (0.864–0.883) (Figure 2A). Meanwhile, a total of 984 real ICH cases were included as the testing set to assess the efficiency of the H-system. The treatment plans automatically output by the H system were compared with the original treatment plans of real ICH cases, the accuracy of the H-system’s treatment plan was 88.55 (88.15–88.94)%, the specificity was 85.71 (84.99–86.43)%, and the sensitivity was 91.83 (91.01–92.65)%. The AUC value of the H-system in the testing set was 0.887 (0.884–0.891) (*p* < 0.001; Table 2; Figure 2B), which means that the treatment output by H-system was accurate and reliable.

**TABLE 2:** Accuracy and AUC of H-system in the testing set.

Group	Accuracy (%) (95% CI)	AUC
H-system	88.55 (88.16–88.94)	0.887 (0.884–0.891)
Plan I	91.83 (91.01–92.65)	
Plan II	85.71 (84.99–86.43)	
Plan IIA	78.03 (76.14–79.92)	
Plan IIB	89.15 (84.40–93.89)	

Furthermore, the accuracy of Plan IIA’s output by the H-system was 85.71 (84.99–86.43)%, which was significantly lower than Plan I’s accuracy of 91.83 (91.01–92.65)% (*p* < 0.05) and Plan IIB’s accuracy of 89.15 (84.40–93.89)%. The different accuracy between Plan IIA and Plan I meant H-system made more mistakes when outputting Plan IIA.

#### Comparison Between Doctors and H-System

To further study the efficiency of the H-system, the comparison between doctors and H-system was performed in two aspects, the total number of cases handled in a fixed time and the total time spent on a fixed number of cases.

##### Efficiency of Doctor and H-System on the Fixed Quantity Cases

A total of 60 real ICH cases were randomly extracted from the N-eEMRD. The mean time that H-system spent on 60 cases was 280.80 ± 3.82 s, and the mean time on a single case was 4.68 ± 0.89 s. For doctors, the mean time Doctor-5Y and Doctor-1Y spent on 60 cases was 10,757.33 ± 94.54 and 19,494.67 ± 121.29 s, and the mean time on a single case was 181.14 ± 19.63 and 324.32 ± 38.44 s, respectively. As shown in Table 3, the time both Doctor-5Y and Doctor-1Y spent is significantly longer as compared with H-system (Figures 2C,D). Furthermore, the sensitivity, specificity, accuracy, and AUC of H-system were 92.86 (83.99–100.00)%, 84.38 (76.61–92.14)%, 88.33 (84.20–92.47)%, and 0.886 (0.0.844–0.928)%, respectively (Table 4). Furthermore, Plan IIA’s accuracy of H-system, Doctor-1Y and Doctor-5Y were 72.73 (50.15–95.31)%, 66.63 (40.40–92.87)%, and 78.79 (65.75–91.83)%, which were significantly lower than that of Plans I and IIB. As shown in Table 4, the AUC of Doctor-1Y is 0.798 (0.777–0.819), which is significantly lower than that of the H-system (*p* < 0.05) (Figure 2E). However, no significant statistical difference in AUC was found between H-system and Doctor-5Y (*p* = 0.225). Then the κ value between H-system and Doctor-5Y was 0.963 (0.938–0.988) (*p* < 0.01; Table 4), indicating a high degree of agreement on treatment plan was found between H-system and Doctor-5Y.

**TABLE 3:** Comparison of time for handling with 60 cases and single case among H-system and doctors.

Group	Time (60 cases)	Time (single)	*p*-Value
H-system	280.80 ± 3.82 s	4.68 ± 0.89 s	…
**Doctors**			
Doctor-1Y	19,494.67 ± 121.29 s	324.32 ± 38.44 s	<0.001
Doctor-5Y	10,757.33 ± 94.54 s	181.14 ± 19.63 s	<0.001

**TABLE 4:** Comparison of efficiency for handling 60 cases among H-system and doctors.

Group	Sensitivity (%) (95% CI)	Specificity (%) (95% CI)	Accuracy (%) (95% CI)	AUC (95% CI)	κ Value (95% CI)
H-system	92.86 (83.99–100.00)	84.38 (76.61–92.14)	88.33 (84.20–92.47)	0.886 (0.844–0.928)	…
**Doctors**					
Doctor–1Y	84.52 (79.40–89.64)	75.00 (67.24–82.77)	79.44 (77.05–81.84)	0.798 (0.777–0.819)	0.827 (0.785–0.868)
Doctor-5Y	94.05 (88.93–99.17)	86.46 (81.99–90.93)	91.51 (87.94–95.08)	0.895 (0.835–0.955)	0.963 (0.938–0.988)

##### Efficiency of Doctor and H-System in a Fixed Time

The number of cases handled by Doctor-1Y and Doctor-5Y in 60 min was compared with H-system, respectively. The mean numbers of cases handled by the H-system were was 766, which was not only significantly greater than that of Doctor-1Y (13 cases) but also significantly greater than that of Doctor-5Y (21 cases) (Figure 2F; *p* < 0.01).

Furthermore, the accuracy of the H-system was 87.47 (86.63–88.31)%, and the specificity and sensitivity were 84.91 (82.81–87.01)% and 90.43 (89.58–91.28)%. For Doctor-1Y, the accuracy was 79.61 (71.34–87.87)%, and the specificity and sensitivity were 74.29 (62.00–86.58)% and 84.92 (81.50–88.34)%, all of which were significantly lower than that of H-system (*p* < 0.01). However, for Doctor-5Y, the accuracy was 89.15 (84.40–93.89)%, and the specificity and sensitivity were 85.05 (74.24–95.86)% and 93.94 (80.90–100.00)%, all of which were not significantly higher than that of H-system (*p* > 0.05; Table 5).

**TABLE 5:** Comparison of cases, sensitivity, specificity, and accuracy in a fixed time among H-system and doctors.

Group	Sensitivity (%) (95% CI)	Specificity (%) (95% CI)	Accuracy (%) (95% CI)	Cases	*p*-Value
H-system	90.43 (89.58–91.28)	84.91 (82.81–87.01)	87.47 (86.63–88.31)	766 (763–769)	…
**Doctor**					
Doctor-1Y	84.92 (81.50–88.34)	74.29 (62.00–86.58)	79.61 (71.34–87.87)	13 (11–15)	<0.01
Doctor-5Y	93.94 (80.90–100.00)	85.05 (74.24–95.86)	89.15 (84.40–93.89)	21 (18–25)	<0.01

## Discussion

In the present study, we designed the H-system to automatically analyze the eEMR of patients with ICH and output the treatment plan. Previously, some researchers have developed models for automated detection of CT scans to assist radiologists (Chen et al., 2021). Although the models showed high accuracy, important clinical symptoms and signs were not included in these studies (Krittanawong et al., 2017; Chilamkurthy et al., 2018; Abedi et al., 2020; Nawabi et al., 2020; Gibicar et al., 2021). To analyze the cases more comprehensively, the H-system can not only recognize the CT reports but also analyze the clinical manifestations. The H-system was founded based on BERT and trained to identify and analyze the eEMR of ICH cases that include medical history, physical examination, and CT report.

### Efficiency of H-System

The accuracy of the H-system’s treatment plan in the testing set was 88.55 (88.16–88.94)%, and the specificity and sensitivity were 85.71 (84.99–86.43)% and 91.83 (91.01–92.65)%. Furthermore, Doctor-1Y and Doctor-5Y were included in this study to test the efficiency of the H-system. For Doctor-1Y, the time spent on 60 cases was significantly longer than that of the H-system, but the accuracy sensitivity, specificity, PPV, and NPV were significantly lower than that of the H-system. For Doctor-5Y, the time spent on 60 cases was also longer than that of the H-system, however, as shown in Table 4, a strong correlation is found between the H-system’s treatment plan and Doctor-5Y’s treatment plan, κ = 0.963 (0.938–0.988). These results indicated that H-system could rapidly and automatically recognize the N-eEMR of ICH and output an accurate treatment plan. Compared with Doctor-1Y, the accuracy of the H-system was more closed to that of Doctor-5Y.

To further test the efficiency of the H-system, the number of cases and accuracy of the H-system and doctors in a fixed time were also calculated and analyzed. For H-system, the mean number of cases handled in 60 min was 766 (763–769), and the accuracy was 87.47 (86.63–88.31)%. For Doctor-1Y and Doctor-5Y, the mean numbers were 13 (11–15) and 21 (18–25), and the accuracy was 79.61 (71.34–87.87)% and 89.15 (84.40–93.89)%. Obviously, the difference in efficiency between doctors and H-system was huge and significant statistically. These results suggested that H-system might provide a reliable way to automatically recognize and analyze medical text data of patients with ICH and output accurate treatment plans with high efficiency. Interestingly, compared with H-system with Doctor-5Y, the accuracy of the H-system was slightly lower, but there was no significant statistical difference between the two groups (*p* = 0.17).

### Development of H-System

The H-system in the study included a pretrained language model BERT for NER and an expert module for logical judgment of extracted entities according to the ICH clinical treatment rules. The expert module consists of output entities imported from the BERT network. First, it matches keywords with their attributes by words segmentation and regular expression in the entities. Then, the expert module carries out weighted arithmetic according to the logic table of the ICH clinical case. Finally, the results of weighted arithmetic are expressed in the form of a weighted score. Meanwhile, a database called N-eEMRD was built up to provide eEMR of cases for training and testing the H-system in this study. The N-eEMRD included eEMRs of simulated ICH cases, which were used for the development and internal validation, and real ICH cases, which were used for external validation and efficiency testing. Although a total of 984 real ICH cases were included in this study, these cases might not cover all possible situations, and the quantity of some special ICH cases was not enough for training the model. Therefore, we designed not only common ICH cases but also rare ICH cases, making the simulated cases have enough quantity and coverage. Similar to the eEMR of real ICH cases, each eEMR of a simulated ICH case consisted of the patient’s general information, medical history, physical examination, and CT report (Supplementary Appendix 2). After analysis of more than 900 real ICH cases and reference of the guidelines in the last 10 years, we designed the clinical and CT manifestations of simulated ICH cases. To exclude the irrational cases, the rationality and logicality of each simulated eEMR were checked by at least two experienced neurosurgeons. Then two neurosurgeons with at least 20 years of working experience analyzed each simulated eEMR and made a treatment plan, which was set as the golden standard for this simulated case (Figure 1B).

It is known that making a proper treatment plan for a critical emergency, such as ICH, is always complicated and challenging. First, information that includes the patient’s general information, medical history, physical examination, and supplementary examination will be collected as detailed as possible in a few minutes. After that, the doctor will comprehensively and carefully analyze this complicated medical information and then make a proper treatment plan immediately. As result, making a proper treatment plan for the patient with ICH is not easy even for a neurosurgeon. Algorithms, which automatically segment and interpret CT images of patients with ICH, can provide vital information about the intracranial conditions, such as the location and volume of hematoma, mass effect, and shifting of middle-line structure (Yang et al., 2018; Nawabi et al., 2020, 2021; Shi et al., 2020). All of these are vital to conduct diagnoses and make proper treatments. However, other vital information, such as the patient’s symptoms and signs, which are mainly obtained from medical history and physical examination, is still needed for a neurosurgeon to make a proper and rapid treatment plan. Therefore, not only the CT results but also the medical history and physical examinations are crucial to making an appropriate treatment plan for patients with ICH. In the present study, neurosurgeons and AI researchers worked together to design the simulated ICH cases and labeled the keywords of ICH eEMR. Furthermore, neurosurgeons designed the rules of making a proper treatment plan and then discussed with AI researchers the logic of the foundation of the model. As shown in the results, the time H-system spent on a single case was only 4.68 ± 0.89 s, and the treatment plan output by H-system was accurate, indicating H-system has the ability to handle a large number of cases in a short time. More importantly, H-system is not like a human who may make mistakes due to negative factors, for instance, fatigue, stress, and mood fluctuations. However, in view of the particularity of clinical medicine, we should be more cautious about the application of AI in clinical events, especially emergency events, such as ICH (Gilvary et al., 2019). For the treatment plan of ICH, the most important things are not only accuracy but also safety and reliability.

### Related Study

Different from most AI systems, which focused on automatic image interpretation, disease phenotyping, and disease prediction (Prevedello et al., 2017; Bacchi et al., 2019; Brugnara et al., 2020; Monteiro et al., 2020), the H-system in this study aimed to automatically analyze the text data of eEMR and provide a treatment plan. Based on BERT, key information was extracted from the medical history, physical examination, and CT report. Therefore, as compared with image interpretation, the H-system could get more critical information from medical text records to make an appropriate treatment plan.

In addition, the model of the H-system can also be used to automatically analyze the medical text record of other diseases. This means the model may be generalized to similar diseases in other neurosurgery diseases. However, before that, the new BERT model and new rules for the expert module need to be designed and trained according to the different diseases.

### Limitations

Although the H-system can automatically recognize and analyze the medical record of ICH (such as medical history, physical examination, and CT results) and output appropriate treatment plans, we have to admit that there are still several limitations in this study. First, the H-system is based on BERT, which still needs more testing for complex cases and complicated clinical application (Lee et al., 2020; Li et al., 2020). Therefore, the H-system would make mistakes when handling some complex cases. We found that the accuracy of Plan IIA was significantly lower than that of Plans I and IIB. After analyzing the eEMRs and golden standard of cases in Plan IIA, we found they were more complicated than other cases and might provide equivocal information on some key points, which might be a challenge even for neurosurgeons. This meant the model needed further optimization and more training. Second, larger sample size is still needed to further validate the model and algorithm of this study. In addition, H-system in this study is designed to identify the eEMR and make a treatment plan according to the eEMR and should be performed in the emergency condition (usually in the first 60 min of the emergency room). Therefore, like human doctors, H-system may also miss some important information that is lost or vacant in the eEMR, which is inevitable in an emergency condition.

## Conclusion

The H-system designed in the study automatically recognizes and analyzes medical text data of ICH patients and rapidly output accurate treatment plans with high efficiency. It may provide a reliable and novel way to automatically and rapidly handle emergency cases, such as ICH.

## Data Availability Statement

The raw data supporting the conclusions of this article will be made available by the authors, without undue reservation.

## Ethics Statement

Ethical review and approval was not required for the study on human participants in accordance with the local legislation and institutional requirements. Written informed consent was obtained from the individual(s), and minor(s)’ legal guardian/next of kin, for the publication of any potentially identifiable images or data included in this article.

## Author Contributions

LJ and JL designed and managed the study. BD, WZ, and JL performed the material preparation, data collection, and analysis. BD and LJ wrote the first draft of the manuscript. QS contributed to data collection and processing. All authors contributed to the study conception, commented on previous versions of the manuscript, and read and approved the final manuscript.

## Conflict of Interest

The authors declare that the research was conducted in the absence of any commercial or financial relationships that could be construed as a potential conflict of interest.

## Publisher’s Note

All claims expressed in this article are solely those of the authors and do not necessarily represent those of their affiliated organizations, or those of the publisher, the editors and the reviewers. Any product that may be evaluated in this article, or claim that may be made by its manufacturer, is not guaranteed or endorsed by the publisher.

## Abbreviations

Doctor-1Y: doctors with 1 year of working experience in neurosurgery
Doctor-5Y: doctors with 5 years of working experience in neurosurgery
eEMR: emergency electrical medical record
N-eEMRD: neurosurgery emergency electrical medical record database
AI: artificial intelligence
ICH: spontaneous intracerebral hemorrhage
NLP: natural language processing
AUC: area under the receiver operating characteristics curve
NPV: negative predictive value
PPV: positive predictive value
ROC: receiver operating characteristic curve.
