# Supplementary material for: Development and Validation of an Automatic System for Intracerebral Hemorrhage Medical Text Recognition and Treatment Plan Output
Source: Front Aging Neurosci. 2022 Apr 8;14:798132. doi: 10.3389/fnagi.2022.798132 (PMC9028758; doi:10.3389/fnagi.2022.798132)
Supplement: Supplementary file 1 [file Table_1.docx]

Appendix 1

1. The format of eEMR

| Medical history | Complains | The most prominent main symptoms |
| --- | --- | --- |
|  | History of present illness | Onset condition of disease, main symptoms, concomitant symptoms and differential symptoms, development and evolution of the disease |
|  | Important history of past illness | Important past illness history which may be related to diagnosis and treatment of ICH |
| Physical examination | Vital signs | Respiration rate, heart rate, blood pressure, oxygen saturation value |
|  | Grade of consciousness and Glasgow score (GCS) | State of consciousness (Ⅰ-Ⅵ grade)  Grade of GCS (3-15 points) |
|  | Pupils changes and limb movements | Symmetry, sizes, and light reflections of bilateral pupils  Movements of body and four limbs |
| CT report | Lateralization analysis of lesion | Left or right |
|  | Location analysis of lesion | Supratentorial lesion  Infratentorial lesion |
|  | Qualitative analysis of lesion | The density of lesion |
|  | Quantitative analysis of lesion | Volume of lesion  Mass effect  Display of important sulcus and cisterns |
