# Supplementary material for: Development and Validation of an Automatic System for Intracerebral Hemorrhage Medical Text Recognition and Treatment Plan Output
Source: Front Aging Neurosci. 2022 Apr 8;14:798132. doi: 10.3389/fnagi.2022.798132 (PMC9028758; doi:10.3389/fnagi.2022.798132)
Supplement: Supplementary file 2 [file Table_2.docx]

Appendix 2

The principles for making treatment plan

The treatment plan was made according to the evaluation of ICH patient’s clinical condition and the latest guideline for ICH, including

(1) Patients’ medical history, including onset condition of disease, main symptoms, concomitant symptoms and differential symptoms, development and evolution of the disease, important past illness history which may be related to diagnosis and treatment of ICH, etc.

(2) Important clinical symptoms and signs, including the symptoms and signs of intracranial hypertension such as headache, nausea and vomiting, Cushing response, state of consciousness (from awake state to severe coma, which correspond toⅠ-Ⅵ grade respectively), Grade of GCS (3-15 points) , sizes, light reflections and symmetry of bilateral pupils, movements of body and four limbs, etc.

(3) CT manifestation, including

1) lateralization analysis of lesion (left side？, right side？)

2) location analysis of lesion (supratentorial lesion?, infratentorial lesion?, subdural hemorrhage?, cerebral parenchymal hemorrhage?, intraventricular hemorrhage?, hemorrhage of basal ganglia?, thalamus?, cerebellar?, brain stem?, etc)

3) Qualitative analysis of lesion (the density of lesion, high density?, Low density?, mixing density?)

4) Quantitative analysis of lesion, including volume of lesion (volume of hematoma, volume of diameter, depth from hematoma to cortical surface, range of cerebral edema, etc), mass effect (compression of brainstem compression of ventricle, hydrocephalus from ventricular obstruction, midline shift, etc), display of important sulcus and cisterns (compression of ambient cistern, basal cistern, sylvian cistern, etc)
