# Supplementary material for: Development and Validation of an Automatic System for Intracerebral Hemorrhage Medical Text Recognition and Treatment Plan Output
Source: Front Aging Neurosci. 2022 Apr 8;14:798132. doi: 10.3389/fnagi.2022.798132 (PMC9028758; doi:10.3389/fnagi.2022.798132)
Supplement: Supplementary file 3 [file Table_3.docx]

**Weight Score for the Treatment Plans**

**1. A weighting score beyond 1.5 is an absolute surgical indication, which means the patient needs immediate emergency surgery.**

**2. A weighting score=1.5 is a relative surgical indication, which means patient does not need emergency surgery for the time being, but his/her condition is not stable and he/she may need surgery if his/her condition deteriorates.**

**3. A weighting score below 1.5 means patient does not need immediate emergency surgery, and his/her condition is relatively stable, which means he/she also unlikely needs surgery in the subsequent time during hospitalization.**

| **CT Performance** |  | **Weighting Score** |
| --- | --- | --- |
| **Hematoma volume [supratentorial hemorrhage (including thalamus, basal ganglia area, frontal lobe, insula, occipital lobe), non-intracerebroventricular, etc]** | **>60ml** | **2.0** |
|  | **>40ml** | **1.5** |
|  | **30ml-40ml** | **1.5** |
| **Hematoma volume (temporal lobe)** | **>20ml-30ml** | **1.5** |
| **Hematoma volume [subtentorial hemorrhage (including cerebellum, brainstem (midbrain, pontine, medulla oblongata), non-intracerebral ventricles, etc]** | **>10ml-15ml** | **1.5** |
|  | **>15ml** | **2.0** |
| **Midline structural shift** | **0.5cm-0.9cm** | **1.5** |
|  | **≥1cm** | **2.0** |
| **Lateral ventricle shape and size** | **Lateral ventricular compression occlusion >1/2** | **1.0** |
|  | **Lateral ventricular dilatation > 1/2 of normal** | **0.5** |
|  | **Lateral ventricular hemorrhage casting** | **0.5** |
| **Shape and size of the fourth ventricles** | **Pressure occlusion >1/2** | **1.0** |
|  | **Dilation > 1/2 of normal** | **0.5** |
|  | **Completely blocked or intraventricular hemorrhage casting** | **1.0** |
| **Triple ventricle** | **Completely blocked or intraventricular hemorrhage casting** | **0.5** |
| **cerebral sulcus and cerebral pool** | **Poor display of ring pool** | **1.0** |
|  |  |  |
| **Clinical manifestations** |  |  |
| **Vital signs** | **When heart rate, blood pressure, and oxygen saturation are zero** | **-∞（Meaningless）** |
| **GCS score** | **12-3** | **When GCS=12, score 0.5. For every 1 point decrease in GCS, add 0.1** |
| **GCS score reduction speed**  **(Green channel process time less than 1 hour)** |  | **For every 1 point decrease in GCS within 30 minutes, add 0.5** |
| **Bilateral pupil size and symmetry of light reflex (exclude non-high cranial pressure factors, such as direct damage to the optic nerve and the oculomotor nerve nucleus)** | **Size asymmetry** | **1.5** |
|  | **Light reflection asymmetry** | **1.5** |
|  | **Size >5mm, light reflection disappears** | **1.5** |
|  | **Bilateral pupil dilatation and fixed** | **1.0** |
|  |  |  |
