# Supplementary material for: Development and Validation of an Automatic System for Intracerebral Hemorrhage Medical Text Recognition and Treatment Plan Output
Source: Front Aging Neurosci. 2022 Apr 8;14:798132. doi: 10.3389/fnagi.2022.798132 (PMC9028758; doi:10.3389/fnagi.2022.798132)
Supplement: Supplementary file 4 [file Table_4.docx]

Table A. An example of eEMR

| Section | Content |
| --- | --- |
| Medical history | 55 years old male, complained sudden headache, nausea and vomiting with left limb hypodynamia for 3 hours, with on special past illness history. |
| Physical examination | P 65 times/min, Bp 190/100mmHg, R 17 times/min, oxygenation 96%, GCS score 10, left pupil diameter 3mm, pupilary light reflex was sensitive, right pupil diameter 3.5mm, pupilary light reflex was dull, the left limbs had no obvious activity under pain stimulation, the right limbs had autonomous activity without pain stimulation, left Babbitt sign (+), right Babbitt sign (-). |
| CT report | CT showed hemorrhage in the right basal ganglia, with obvious space occupying effect. The volume of hematoma was about 50ml, and the midline structure shifted to the left by about 1.5cm. The right ventricle was obviously compressed by about 1/3 as compared with the left ventricle. |

Table B. Key words extracted from the eEMR

| Words | Section |
| --- | --- |
| headache | Medical history |
| nausea | Medical history |
| vomiting | Medical history |
| hypodynamia | Medical history |
| P 65 times/min | Physical examination |
| Bp 190/100mmHg | Physical examination |
| R 17 times/min | Physical examination |
| oxygenation 96% | Physical examination |
| GCS score 10 | Physical examination |
| left pupil diameter 3mm | Physical examination |
| pupilary light reflex | Physical examination |
| sensitive | Physical examination |
| right pupil diameter 3.5mm | Physical examination |
| dull | Physical examination |
| left limbs | Physical examination |
| no obvious activity | Physical examination |
| pain stimulation | Physical examination |
| right limbs | Physical examination |
| autonomous activity | Physical examination |
| left Babbitt sign (+) | Physical examination |
| right Babbitt sign (-) | Physical examination |
| hemorrhage | CT report |
| right basal ganglia | CT report |
| obvious space occupying effect | CT report |
| volume of hematoma was about 50ml | CT report |
| the midline structure shifted to the left by about 1.5cm | CT report |
| right ventricle was obviously compressed by about 1/3 as compared with the left ventricle | CT report |
